# Viewport prediction with cross modal multiscale transformer for 360° video streaming

**DOI:** 10.1038/s41598-025-16011-7

**Published:** 2025-08-19

**Authors:** Yangsheng Tian, Yi Zhong, Yi Han, Fangyuan Chen

**Affiliations:** 1 School Of Information Engineering, Wuhan University of Technology, LuoShi Road, Wuhan, 430070 Hubei China; 2 Wuhan Polytechnic University, ChangQing Road, Wuhan, 430023 Hubei China

**Keywords:** 360^◦^ video streaming, Viewport prediction, Transformer structure, Multi modal, Computer science, Information technology

## Abstract

In the realm of immersive video technologies, efficient 360° video streaming remains a challenge due to the high bandwidth requirements and the dynamic nature of user viewports. Most existing approaches neglect the dependencies between different modalities, and personal preferences are rarely considered. These limitations lead to inconsistent prediction performance. Here, we present a novel viewport prediction model leveraging a Cross Modal Multiscale Transformer (CMMST) that integrates user trajectory and video saliency features across different scales. Our approach outperforms baseline methods, maintaining high precision even with extended prediction intervals. By harnessing the Cross Modal attention mechanisms, CMMST captures intricate user preferences and viewing patterns, offering a promising solution for adaptive streaming in virtual reality and other immersive platforms. The code of this work is available at https://github.com/bbgua85776540/CMMST.

## Introduction

Video has become a popular medium for socializing and sharing, with 360-degree video offering richer content that delivers a more immersive and engaging experience. In recent years, leading platforms like YouTube and Facebook have been actively promoted 360-degree video services. In particular, virtual reality is a key technology in the new conceptual metaverse that has recently emerged, and 360-degree video is one of its key distribution media^1^. Unlike traditional videos, 360-degree videos usually require very high video resolution (usually $\:\ge\:$4 K) to ensure satisfactory Quality of Experience (QoE). While mainstream platforms recommend a minimum connection speed of 25 Mbps for UHD streaming^2^, the 2021 global average bandwidth per user reached only 190.1 kbps^3^. This disparity underscores the critical need for research into efficient 360-degree video transmission.

**Fig. 1:**
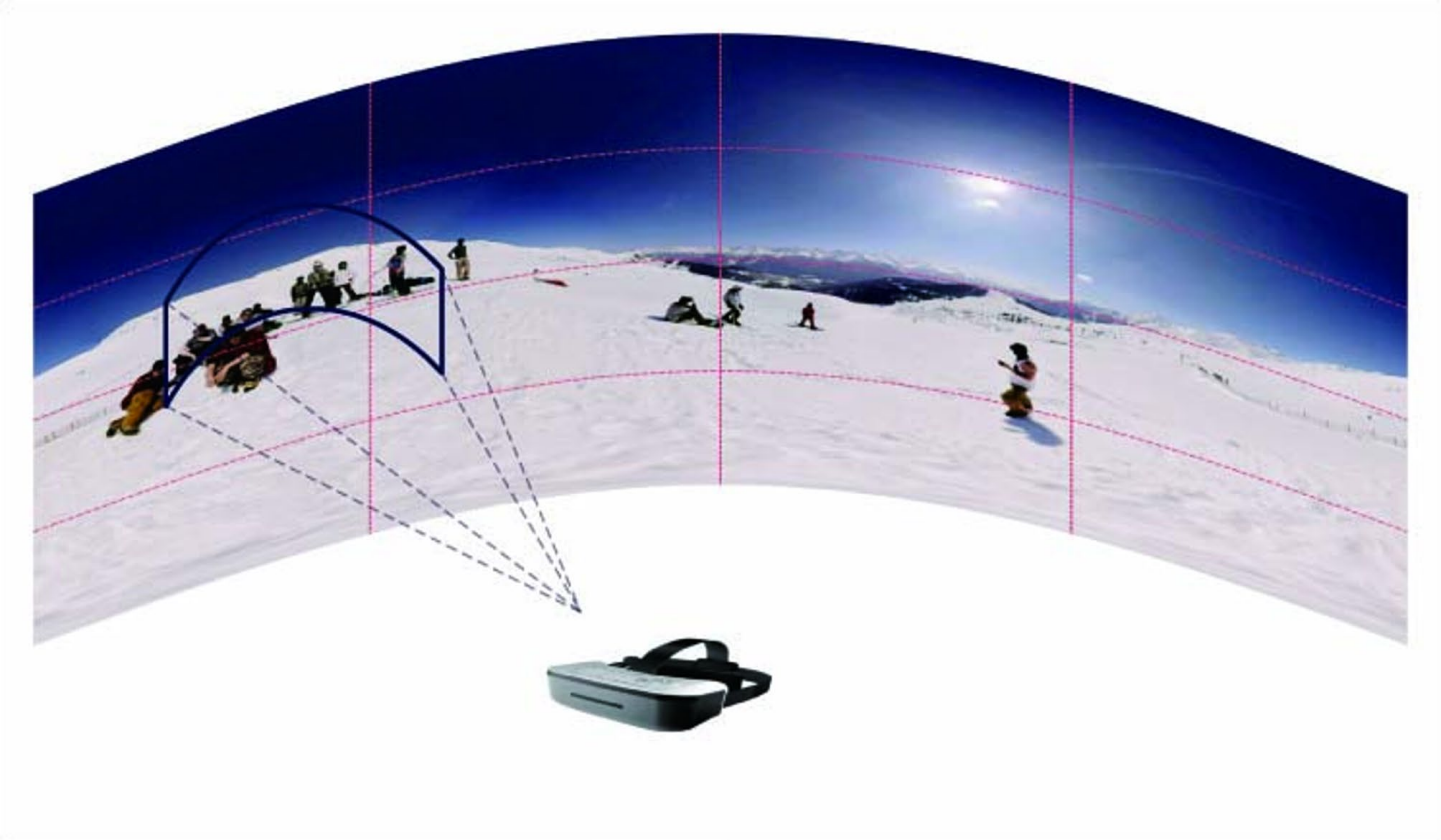
An illustration of the tile-based 360-degree video streaming, dividing the video rectangular frame into RxC tiles area, where *R* = 4, C = 4.

The established solution to bandwidth constraints is selective streaming^4^. Leveraging the standardized MPEG-DASH protocol^5^, 360-degree videos can be adaptively streamed similarly to traditional video content. As illustrated in Fig. 1, users typically observe only a limited portion of the 360-degree environment at any given time, leaving most areas unviewed and resulting in significant bandwidth waste. Current approaches spatially partition 360-degree videos into multiple non-overlapping rectangular regions (tiles)^6,7^, then temporally segment them into fixed-duration chunks. This allows high-quality encoding only for viewport-projected tiles, while other regions are transmitted at lower quality. However, this technique requires predictive chunk requests to accommodate network latency. Viewport prediction remains challenging due to dynamic viewport changes—incorrect predictions directly degrade QoE^8^. The complexity stems from multiple factors: while some studies model user preferences through head movement trajectories and video content analysis, accurate viewport prediction continues to pose significant research challenges.

Existing methods typically model user preferences using a single modality, often neglecting dependencies between different modalities and multiscale features. To address these limitations, we propose CMMST (Cross Modal Multiscale Transformer), a novel viewport prediction model based on the Transformer architecture^9^. Our model predicts users’ future viewports by jointly analyzing historical head trajectories and video content. The proposed approach first projects image saliency feature maps and user viewing trajectories into a shared embedding space, then captures spatio-temporal dependencies at multiple scales through attention mechanisms to effectively model user preferences. We evaluate CMMST on two benchmark datasets, with experimental results demonstrating superior viewport prediction accuracy compared to baseline methods.

## Related work

The tile-based adaptive 360-degree video streaming algorithm utilizes head-mounted displays (HMDs) to rapidly respond to viewer head movements. Viewport prediction algorithms play a vital role in optimizing 360-degree video streaming. However, predicting future viewports remains challenging as they depend on multiple factors, including user preferences, head movement velocity, and video content characteristics.

Previous work^10^ has employed regression methods to predict user viewports by analyzing historical trajectory data. However, these approaches are limited to using only historical trajectory information. Fang et al.^11^ applied a Transformer architecture to model temporal dependencies for long-term viewport scanpath prediction. Additionally, the short prediction window restricts these methods to maintaining only a short streaming buffer, making them often inadequate for complex network conditions. Since only the user’s head movement history is utilized, these methods lack additional contextual information. Consequently, they cannot adapt to individual user preferences and are limited to making effective predictions within short time windows, often failing to accommodate changing video scenarios.

Work^12^ uses clustering to improve the performance of prediction methods by utilizing cross-user viewing behavior information. Despite improvements in prediction accuracy and time window length, these methods require pre-acquisition of cross-user trajectory information for the corresponding video content, which is not accessible in many scenarios. Examples include new videos that have not yet been viewed and live video streams, demonstrating significant limitations of such methods.

Introducing more information to content-aware viewport prediction can indeed greatly improve prediction performance. The temporal and spatial salience of the video, along with historical trajectory data, constitute the three key factors for viewport prediction. Study^13^ used CNNs to capture saliency features and LSTMs to extract historical trajectory features respectively, then predicted future viewports by fusing these two features. However, the features from these different modalities were not effectively fused, resulting in prediction performance inferior to more advanced methods. Chopra et al.^14^ suggests that the user’s viewport depends on the main objects in the video, predicted using multi-target detection algorithms to identify objects in video frames, combined with target tracking and the user’s historical viewport data. However, these detection algorithms can only identify foreground objects in video frames, failing to effectively recognize potentially interesting background elements. Study^15^ performs viewport prediction by categorizing tiles, but ignores individual user uniqueness, as preferences and viewports may vary significantly in content-rich 360-degree videos.

To overcome these limitations, we propose the Cross Modal Multiscale Transformer (CMMST). The CMMST projects both the viewport and 360-degree video content into uniformly-sized rectangular frames, enabling effective Cross Modal feature alignment. This unified representation facilitates dependency extraction between visual content and user behavior modalities. We validate our model on benchmark datasets and experimentally demonstrate that it outperforms baseline approaches.

## Method

We formulate the 360-degree video streaming viewport prediction problem as follows: Given a series of 360-degree video frames $\:{F}_{1:T}=\left\{{f}_{1},{f}_{2},\dots\:{f}_{\text{t}}\dots\:,{f}_{T}\right\}$, where $\:{f}_{t}\in\:{\mathbb{R}}^{H\times\:W}$ corresponds to frame t of the video. The orientation of the user’s head on the corresponding frame is $\:{P}_{1:T}^{i}=\left\{{p}_{1}^{i},{p}_{2}^{i},\dots\:{p}_{t}^{i},\dots\:,{p}_{T}^{i}\right\}$, $\:{p}_{t}^{i}$ corresponds to the head orientation of the user i when viewing frame t, where $\:{p}_{t}^{i}=\left({q}_{w},{q}_{x},{q}_{y},{q}_{z}\right)$, $\:{p}_{t}^{i}$ is unit quaternions representation of head orientation with respect to a fixed reference point. In order to construct the dependence of the user trajectory and the video saliency map, we project the user’s historical head movement trajectory onto the corresponding pixel point location of the video frame, i.e. the center of the user’s viewport. Thus, the motion recordings of the user head are converted into viewing trajectories in rectangular video frames. We project the head orientation quaternion to a 2D coordinate $\:{\text{L}}_{\text{t}}^{\text{i}}=\left({\text{x}}_{\text{t}},{\text{y}}_{\text{t}}\right)$, which corresponds to the viewport position in the corresponding video frames. According to the tile-based 360-degree video streaming framework, we divide the video frames into $\:\text{C}\text{o}\text{l}\times\:\text{R}\text{o}\text{w}$ tile regions on average for selective transmission. Varying the tiling of the video can produce different numbers of tiles; more tiles mean less error tolerance in the viewport prediction. To be fair, we use the same tile strategy $\:(Col=16,\:\:Row=9)$ as the baseline method PanoSalNet^16^. According to the viewport coordinate $\:{\text{L}}_{\text{t}}^{\text{i}}=\left({\text{x}}_{\text{t}},{\text{y}}_{\text{t}}\right)$, We can get the index of the tile $\:{\text{M}}_{\text{t}}^{\text{i}}=\left(\text{i}\text{d}{\text{x}}_{\text{c}},\text{i}\text{d}{\text{x}}_{\text{r}}\right)$, where $\:\text{i}\text{d}{\text{x}}_{\text{c}}=\left(\text{W}\times\:16\right)/{\text{x}}_{\text{t}}$, $\:\text{i}\text{d}{\text{x}}_{\text{r}}=\left(\text{H}\times\:9\right)/{\text{y}}_{\text{t}}$. The goal of our viewport prediction model is that, based on the viewport trajectory of the past 1 s $\:{\text{P}}_{1:\text{b}}^{\text{i}}=\left\{{\text{p}}_{1}^{\text{i}},{\text{p}}_{2}^{\text{i}},\dots\:,{\text{p}}_{\text{b}}^{\text{i}}\right\}$, and video metadata from one second ago to n seconds in the future $\:{\text{F}}_{1:\text{T}}=\left\{{\text{f}}_{1},{\text{f}}_{2},\dots\:{\text{f}}_{\text{t}}\dots\:,{\text{f}}_{\text{T}}\right\}$. Predict the tile index of the viewport for the next n seconds $\:{\text{M}}_{\text{t}}^{\text{i}}=\left(\text{i}\text{d}{\text{x}}_{\text{c}},\text{i}\text{d}{\text{x}}_{\text{r}}\right)$. Here b denotes the number of frames in one second of video (i.e., the video frame rate).

## Dataset

**Fig. 2:**
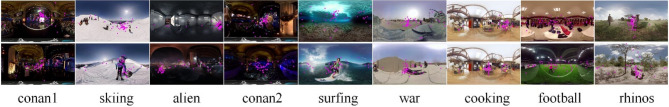
Example frames of dataset, each red dot indicates the center of the user’s viewport.

We employed two widely used datasets encompassing 360-degree videos of diverse categories and corresponding head movement trajectory logs. The first dataset (ds1)^17^ contains nine popular 360-degree videos in four categories, watched by 48 users with an average viewing time of 164 s. The second dataset (ds2)^18^ contains trajectory records of 59 users freely watching five 360-degree videos, each recorded for 70 s. In both datasets, each trace of the head tracking log for both datasets consists of the user’s head position (unit quaternion (w, x, y, z)) along with a timestamp, which is projected to equirectangular frames. Example frames of dataset are shown in Fig. 2, where red dots indicate viewport centers. For videos containing salient objects, while most viewports focus on these regions, numerous outliers exist. For videos without salient objects, viewport distributions are more dispersed.

**Fig. 3:**
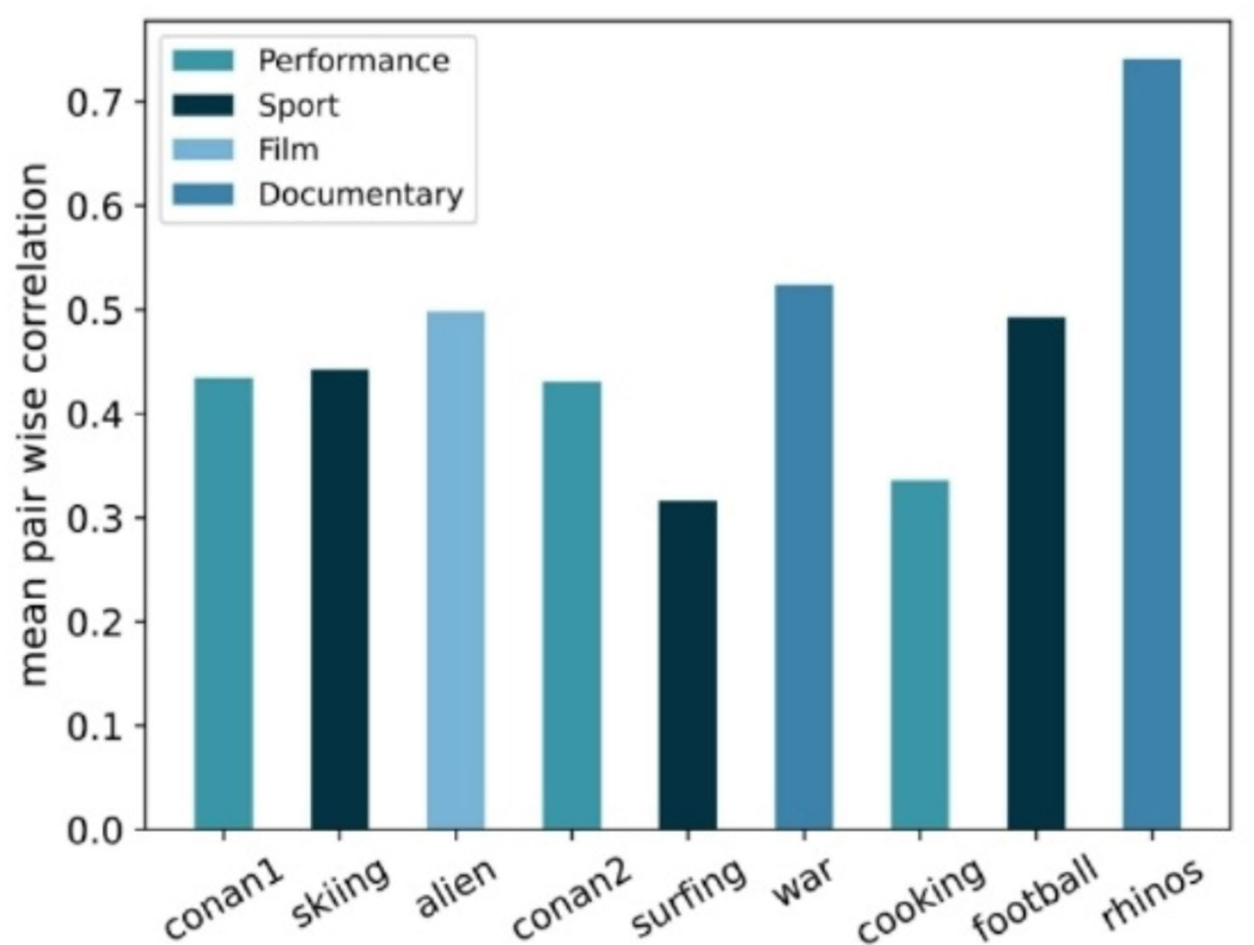
Pair-wise correlation of each video in dataset. Where the colour of the column indicates the video category.

We performed correlation analysis of pairwise user trajectories for each video. The average pairwise Pearson correlation coefficients for each video are shown in Fig. 3. The results clearly demonstrate significant differences in user behavior across different videos. User behavior shows greater consistency for videos with centralized salient regions (e.g., videos featuring rhinos). For performance videos such as football games, which contain rich visual content, users tend to focus on diverse areas. These findings highlight that modeling diverse user preferences is essential for improving viewport prediction accuracy.

## Network overview

**Fig. 4:**
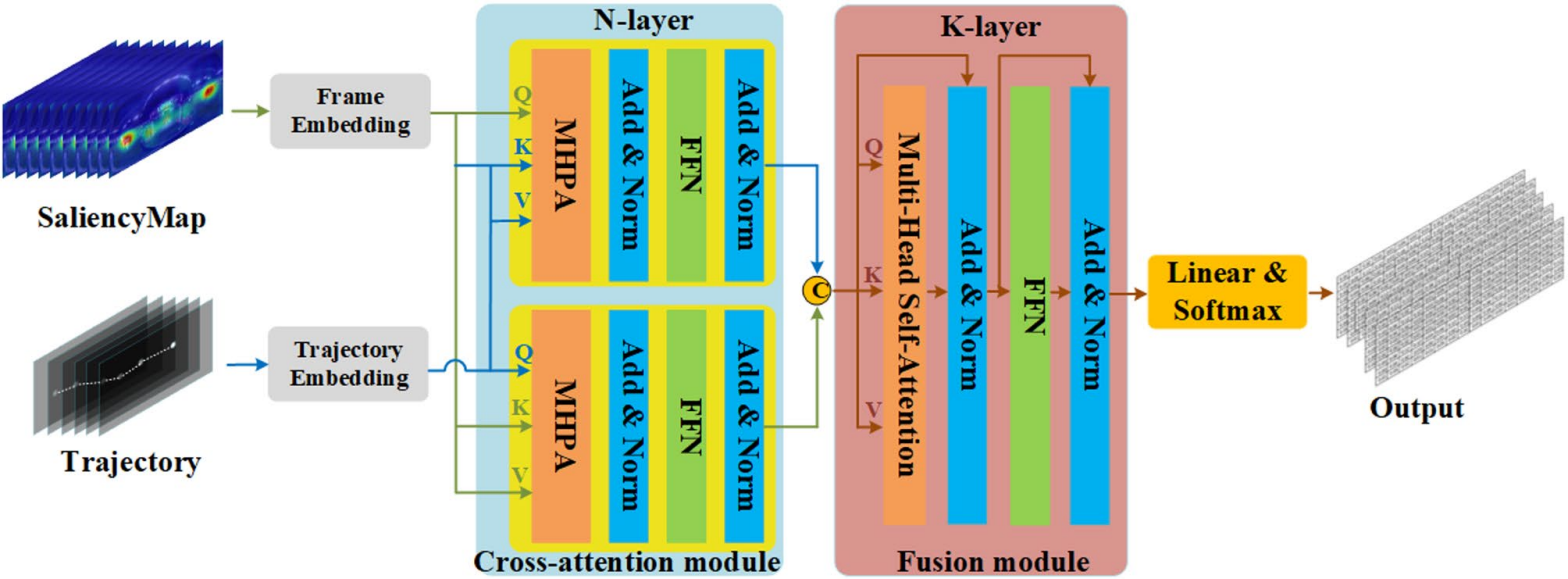
Structure overview of Cross Modal Multiscale Transformer (CMMST). Input saliency map and user trajectory, CMMST output the probability matrix of each tile. MHPA: Multi-Head Pooling Attention; FFN: Feed-Forward Network; “C” denotes the concatenation operation;.

The architecture of our Cross Modal Multiscale Transformer (CMMST) is illustrated in Fig. 4. Our model takes as input both the saliency map and the user’s trajectory, embedding each into a unified vector space compatible with the Transformer architecture. To model user preferences, we employ cross-attention mechanisms to capture relationships between user trajectories and saliency maps. Since video processing typically requires substantial computational resources, we improve efficiency through down-sampling in our Multi-Head Pooling Attention module. This approach achieves three key benefits: (1) enabling progressive spatio-temporal resolution changes, (2) extracting multi-scale spatio-temporal features, and (3) significantly enhancing computational efficiency.

### 360-degree video saliency detection

**Fig. 5:**
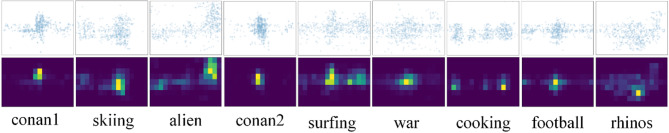
Accumulate fixations and saliency maps of each video. Accumulate fixation maps are in first line, accumulate saliency maps are in second line.

Saliency detection has long been an important research area in visual attention prediction for image and video viewing. Users are more likely to stay in regions of interest^19^. In 360-degree videos, saliency information shows strong correlation with user viewing trajectories^20^, making video saliency features valuable for viewport prediction. While we cannot directly access users’ future viewports, we can infer potential areas of interest through video content analysis. Building upon pioneering work in 360-degree video saliency detection, we employ PanoSalNet^16^ to extract frame-level saliency features, simplifying our problem formulation. Figure 5 presents the accumulated saliency and fixation maps, revealing distinct spatial exploration patterns across different videos. These patterns are encoded as weight matrices, where higher values in the saliency feature matrices indicate regions more likely to attract user attention.

### Cross modal multiscale transformer

Predicting upcoming viewports from user head motion trajectories and video content is a challenging task, particularly when building dynamic systems that integrate two distinct modalities: video saliency features and user head motion features. To accommodate diverse user preferences and address the complexity and variability of network conditions, models must efficiently capture Cross Modal dependencies over extended time periods—a capability overlooked in many previous studies.

#### Frame embedding and trajectory embedding

The standard Transformer^9^ receives as input a 1D sequence of token embeddings. However, our inputs (e.g., video saliency heatmaps) are 3D matrices. To process 360-degree video salience map and user trajectory, we first partition each $\:T\times\:\:H\times\:W$ saliency map (where T represents frame count, H denotes height, and W indicates width) into non-overlapping cubes of size $\:T{\prime\:}\times\:\:H{\prime\:}\times\:W{\prime\:}$. We then apply a linear layer to each cube, equivalent to a 3D convolution with matching kernel and stride sizes, projecting them into the Transformer’s latent dimension D. Formally, we reshape the heatmap $\:{S}_{1:T}\in\:{\mathbb{R}}^{T\times\:H\times\:W}$ into a sequence of flattened 3D cube $\:{X}_{1:T}\in\:\:{\mathbb{R}}^{N\times\:\left(T{\prime\:}H{\prime\:}W{\prime\:}\right)\times\:D}$, where $\:(T,\:H,\:W)$ is the frames, height and width of the 360-degree video salience map. $\:(T^{\prime},\:H^{\prime},\:W^{\prime})\:$is the frames, height and width of each video salience map cube, r is the sample rate on time, and $\:N=THW/rH{\prime\:}W{\prime\:}$.

For trajectory embedding, we apply the same cubing operation to the user’s historical trajectory $\:{L}_{1:b}\in\:{\mathbb{R}}^{b\times\:H\times\:W}$, mapping it to cube embedding features. These features are then fused with corresponding video saliency heatmaps $\:{S}_{1:b}\in\:{\mathbb{R}}^{b\times\:H\times\:W}$ through element-wise multiplication. To capture Cross Modal dependencies, we concatenate the fused hybrid features with future video saliency heatmaps, converting the combined representation into Transformer tokens. Finally, we extract Cross Modal dependencies using multi-head pooling attention.

#### Multiscale cross-attention module

**Fig. 6:**
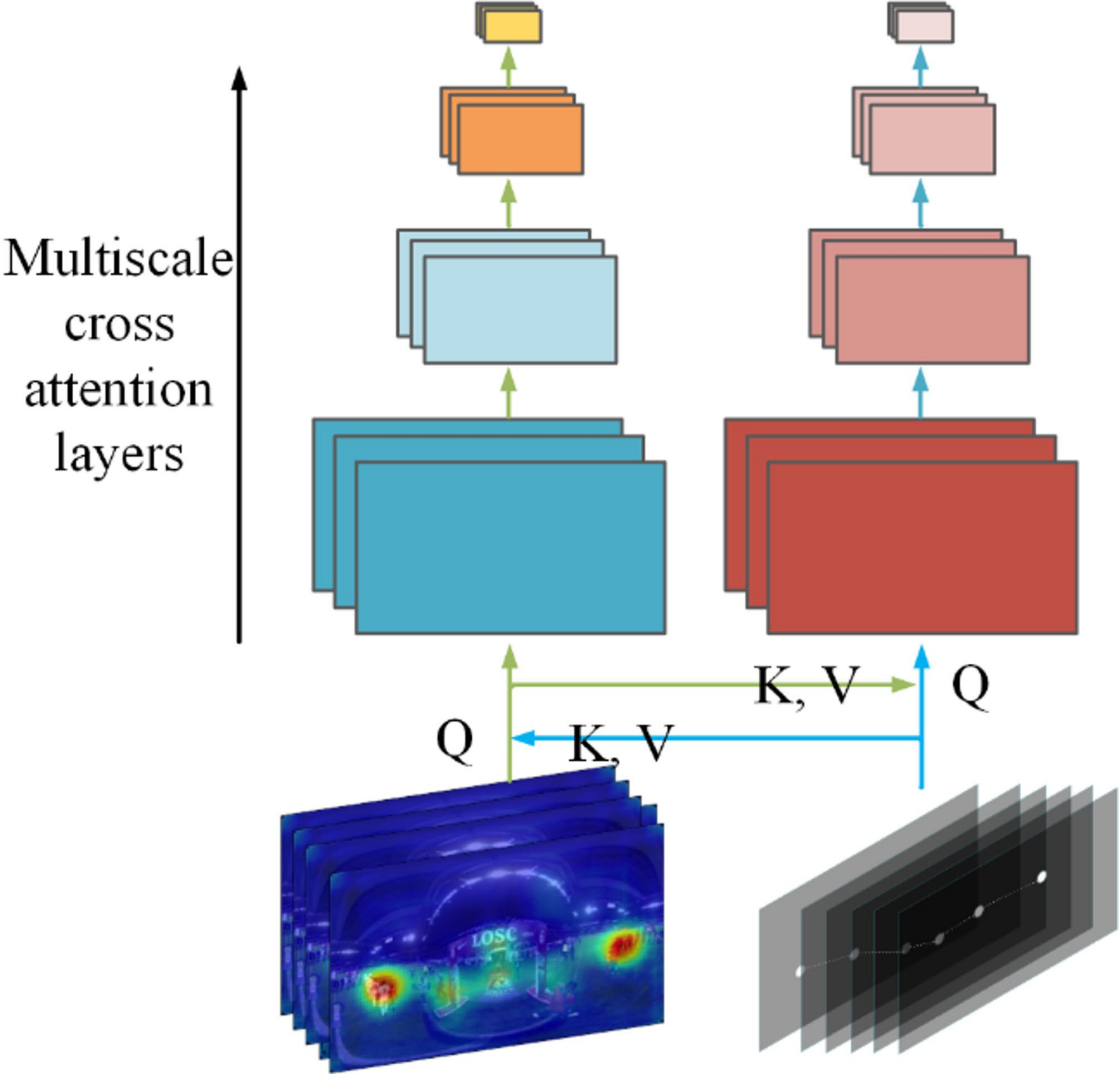
Schematic of the computational loop for Multiscale Cross-Attention Module.

Multi-head grouping attention is a self-attention operation that enables flexible resolution modeling in transformer blocks^21^. This mechanism allows our Cross Modal multiscale Transformer to operate at progressively changing spatio-temporal resolutions. Figure 6 shows the schematic diagram of the multiscale cross-attention module. Specifically, multi-head pooling attention combines sequences of latent tensors to reduce the input sequence length (resolution) while preserving essential features.

Similar to Multi Head Attention for traditional transformers, for a D-dimensional, input vector of length $\:\text{L}$ ($\:L=N\times\:\left(thw\right)$),$\:{X}_{1:t}\in\:\:{\mathbb{R}}^{L\times\:D}$.We then project the input tensor to the intermediate query tensor $\:Q\in\:\:{\mathbb{R}}^{L\times\:D}$ via a $\:D\times\:D$ Linear operation. key tensor $\:K\in\:\:{\mathbb{R}}^{L\times\:D}$ and value tensor $\:V\in\:\:{\mathbb{R}}^{L\times\:D}$.

1 $$\:\begin{array}{c}Q=X{W}_{Q},\:\:K=X{W}_{K},\:\:V=X{W}_{V}\end{array}$$

The pooling attention operation is applied to all intermediate vectors and the convolution operation is performed on $\:\text{Q}$, $\:\text{K}$ and $\:\text{V}$,with chosen convolution kernels k, stride s and padding p. By controlling the parameter $\:{\uptheta\:}(\text{k},\text{s},\text{p})$, the spatial resolution is progressively reduced, reducing the length of the sequence and focusing on shorter vectors, pooling attention is computed as:

2 $$\:\begin{array}{c}PA\left(\bullet\:\right)=Softmax\left(\frac{Conv\left(Q;{\theta\:}_{Q}\right){Conv\left(K;{\theta\:}_{K}\right)}^{T}}{\sqrt{d}}\right)Conv\left(V;{\theta\:}_{V}\right)\end{array}$$

Where the $\:\sqrt{d}$ is normalizing the inner product matrix row wise.

#### Multiple heads

As in^9^ the computation can be parallelized by considering h heads where each head is performing the pooling attention on a non-overlapping subset of $\:D/h$ channels of the D dimensional input tensor X.

Transformer tokens are fed into alternating layers of blocks consisting of multiple pooled attention and MLP (Multi-Layer Perceptron) to extract global attention. Blocks are computed as:

3 $$\:\begin{array}{c}{X}_{1}=MHA\left(LN\left(X\right)\right)+X\end{array}$$

4 $$\:\begin{array}{c}Blocks\left(X\right)=MLP\left(LN\right({X}_{1}\left)\right)+{X}_{1}\end{array}$$

It is worth noting that since tokens contain features from multiple time points, the block layers can extract both (1) temporal dependencies across different frames and (2) spatial dependencies within frames. Finally, considering 360-degree video resolution characteristics, we employ Linear and Softmax layers to map the last frame features into a $\:16\times\:9$ matrix, obtaining the predicted probability for each region $\:{\widehat{M}}_{T}\in\:\:{\mathbb{R}}^{16\times\:9}$. We use Mean Squared Error (MSE) loss to measure viewport prediction accuracy, as it demonstrates higher sensitivity to outliers. The loss function is computed as:

5 $$\:\begin{array}{c}Loss=\frac{1}{T}\sum\:_{i=1}^{T}{\left({\widehat{M}}_{i}-{M}_{i}\right)}^{2}\end{array}$$

where T is the total number of frames predicted, $\:{\widehat{M}}_{i}$ is the predicted probability matrix for each viewport tile in the i-th frame, and $\:{M}_{i}$ is the corresponding ground truth binary matrix.

## Experiments

### Experimental setting

#### Metrics

The Accuracy and Manhattan Error of head movement prediction are used as the evaluation metric. Accuracy is calculated based on the ratio of the number of overlapping tiles between the predicted and ground truth head orientation map to the total number of predicted and viewed tiles. This accuracy metric is averaged across all frames and all users for each video in the dataset.

Tile error denotes the minimum Manhattan distance between the actual tile and the predicted tile, averaged over the video length. The Manhattan Error is reported as average over all frames and over all users for the video. Manhattan Error is computed as:

6 $$\:\begin{array}{c}ManhattanTileError=\frac{\sum\:\left|{L}_{k}-{L}_{k}^{pred}\right|}{T}\end{array}$$

Quality of Experience (QoE) metrics: User-perceived quality is quantitatively assessed through multiple QoE metrics, which we define to empirically evaluate our model’s performance.

The first QoE metric ($\:{Q}_{1}$) is the average viewport bitrate, reflecting the user’s perceived video quality. Given an R × C tiled video with a viewport size of $\:{P}_{w}\times\:{P}_{h}$. For chunk x, $\:{Q}_{1}^{x}$ is denoted as

7 $$\:\begin{array}{c}{Q}_{1}^{x}=\frac{1}{{n}_{x}}{\sum\:}_{i=1}^{{f}_{x}}\frac{\sum\:\:{a}_{c,r}^{i}{B}_{c,r}^{x}}{{tiles}{\left({P}_{w}\right)}_{n}\times\:{tiles}{\left({P}_{h}\right)}_{n}}\end{array}$$

where, $\:{f}_{x}$ is the number of frames in the chunk x. $\:{B}_{c,r}^{x}$ is the bitrate allocated to tile $\:(c,r)$ in chunk x, while $\:{a}_{c,r}^{i}\in\:\left\{\text{0,1}\right\}\:$indicates whether the tile is inside the $\:i-th$ frame’s viewport. $\:tiles{\left({P}_{c}\right)}_{n}\times\:tiles{\left({P}_{r}\right)}_{n}$ represents the total number of tiles displayed in the video player. The normalization constant $\:{n}_{x}$ is derived from the count of unique viewport tiles in chunk x.

The second QoE metric ($\:{Q}_{2}$) quantifies bitrate variation within the viewport across frames. This metric minimizes bitrate fluctuations among tiles in the viewport. For chunk x,

8 $$\:\begin{array}{c}{Q}_{2}^{x}=\frac{1}{{n}_{x}}{\sum\:}_{i=1}^{{f}_{x}}StdDev\left\{{B}_{c,r\:}^{x}:x\in\:tiles{\left({P}_{w}\right)}_{n},\:\:y\in\:{tiles}{\left({P}_{h}\right)}_{n}\right\}\end{array}$$

The third QoE metric ($\:{Q}_{3}$) measures inter-frame bitrate variation within a chunk. This metric aims to minimize quality fluctuations between consecutive viewports (e.g., frame f₁ to f₂). For chunk x,

9 $$\:\begin{array}{c}{Q}_{3}^{x}=\frac{1}{{n}_{x}}{\sum\:}_{i=1}^{{f}_{x}}StdDev\left\{\frac{\sum\:\:{a}_{c,r}^{i}{B}_{c,r}^{x}}{{tiles}{\left({P}_{w}\right)}_{n}\times\:{tiles}{\left({P}_{h}\right)}_{n}}:{a}_{c,r}^{i}=1;\forall\:i\in\:{f}_{x};c\in\:C,r\in\:R\right\}\end{array}$$

The fourth QoE metric ($\:{Q}_{4}$) quantifies inter-chunk viewport bitrate variation. Minimizing this variation is crucial for ensuring consistent Quality of Experience. For chunk x,

10 $$\:\begin{array}{c}{Q}_{4}^{x}=\left|{Q}_{1}^{x}-{Q}_{1}^{x-1}\right|\end{array}$$

For the entire video comprising chunks x, we evaluate the overall QoE through averaged per-chunk measurements, expressed as:

11 $$\:\begin{array}{c}QoE=\frac{1}{X}\left(\sum\:_{x=1}^{X}\left({Q}_{1}^{x}-{Q}_{2}^{x}-{Q}_{3}^{x}\right)-\sum\:_{x=2}^{X}{Q}_{4}^{x}\right)\end{array}$$

#### Baseline

We compare our model with three baseline methods: (1) PARIMA^14^, which extracts motion trajectories of main video objects and predicts them using regression (implemented using publicly available code1 (https://github.com/sarthak-chakraborty/PARIMA (Access Nov 17, 2022))); (2) PanoSalNet^16^, employing an LSTM architecture to process saliency maps and head movement data for viewpoint prediction (using official public code2 (https://github.com/phananh1010/PanoSalNet.git (Access: March 2, 2021))); and (3) Cluster^22^, which groups users based on viewport history and performs predictions via quaternion extrapolation (implemented with code1 using a 1-second prediction window).

#### Training configuration

We train our network using the AdamW optimizer ($\:{\beta\:}_{1}=0.9$, $\:{\beta\:}_{2}=0.999$) with a learning rate of 0.00025, momentum 0.95, weight decay 0.0005, and batch size 16. Following^16^, we partition the dataset^17^ using 5 of 9 videos for training and 4 for validation. For each video, we select one segment with a length of 20–45 s. The video segment is selected so that it contains one or more events that introduce new salient regions and cause the user’s head to be moved fast. The default prediction window is set to be 1 s. The default Multiscale cross-attention layer number N is set to be 4. The default Fusion layer number K is set to be 4. We resize the saliency map to $\:256\times\:144$ to reduce the computation. The final model spent 3 h training 10 epochs on a 4090 GPU. Early stopping criterion: Validation accuracy did not improve for 3 epochs.

## Main result

We conducted statistical significance tests across five independent runs with different random seeds. As shown in Table 1, CMMST demonstrates significant accuracy improvements over baseline methods. PARIMA operates by extracting motion trajectories of dominant video objects and performing regression-based predictions. Although we tested it using shorter video segments containing salient events, PARIMA requires an adaptation period for new input data, ultimately degrading its performance. We evaluated our proposed model on Dataset 2 (ds2), with results presented in Table 2. Using a 1-second prediction window across five independent runs with different random seeds, CMMST achieves an average precision of 0.662 ± 0.0021 (mean ± SD). The model maintains strong performance on this dataset, demonstrating robust generalization capabilities across diverse data scenarios.

To validate the practical efficacy of our viewport prediction framework, we implemented an end-to-end streaming pipeline. This experimental setup enables quantitative evaluation of both bandwidth efficiency and perceptual quality through four QoE metric. Benchmark results demonstrate our method achieves superior QoE compared to baseline methods. These QoE gains substantiate our hypothesis that spatial-temporal attention modeling enables more accurate viewport anticipation, thereby reducing wasteful bandwidth allocation and improving rendered quality where users actually look.

**Table 1:** Comparision with baseline methods on dataset 1.

Model	Accuracy	Tile errors	QoE
PARIMA	0.206	2.741	0.643
PanoSalNet	0.318	1.54	0.741
Clust	0.283	1.169	0.611
CMMST	**0.68 ± 0.0022**	**0.493 ± 0.0055**	**1.18 ± 0.0010**
CMMST-T	0.664 ± 0.0029	0.653 ± 0.057	1.17 ± 0.002

**Table 2:** Comparision with baseline methods on dataset 2.

Model	Accuracy	Tile errors	QoE
PARIMA	0.259	2.881	0.739
PanoSalNet	0.147	2.939	0.566
Clust	0.273	1.191	0.596
CMMST	**0.662 ± 0.0021**	**0.423 ± 0.003**	**0.98 ± 0.0003**

### Ablation experiments of different modes and multiscale pooling attention

To evaluate the contribution of individual modalities, we developed the CMMST-T variant that exclusively processes user trajectory data. Table 1. demonstrates that integrating visual saliency information yields a 0.016 improvement in prediction accuracy while reducing average tile error by 0.16, confirming the complementary value of multimodal inputs.

To rigorously validate the contribution of our multiscale design, we have conducted comprehensive ablation experiments as follows: a) *CMMST-Small*: Only uses fine-scale attention (patch size = 32 × 20).

b) *CMMST-Large*: Only uses coarse-scale attention (patch size = 16 × 10). As shown in Table 3, the complete CMMST maintains no accuracy drop compared to the large variant while requiring only 71% of its FLOPs, establishing an optimal accuracy-efficiency tradeoff.

**Table 3:** Ablation experiments of multiscale pooling attention on dataset 1.

Model	Accuracy	Tile errors	QoE	Acticvations	FLOPs
CMMST	0.68 ± 0.0022	0.493 ± 0.0055	1.18 ± 0.0010	115.5 M	24.9G
CMMST-S	0.672 ± 0.0027	0.525 ± 0.0042	1.17 ± 0.0007	92.25 M	22G
CMMST-L	0.68 ± 0.0036	0.494 ± 0.0097	1.18 ± 0.0008	347.4 M	88.8G

### Impacts of prediction window

**Fig. 7:**
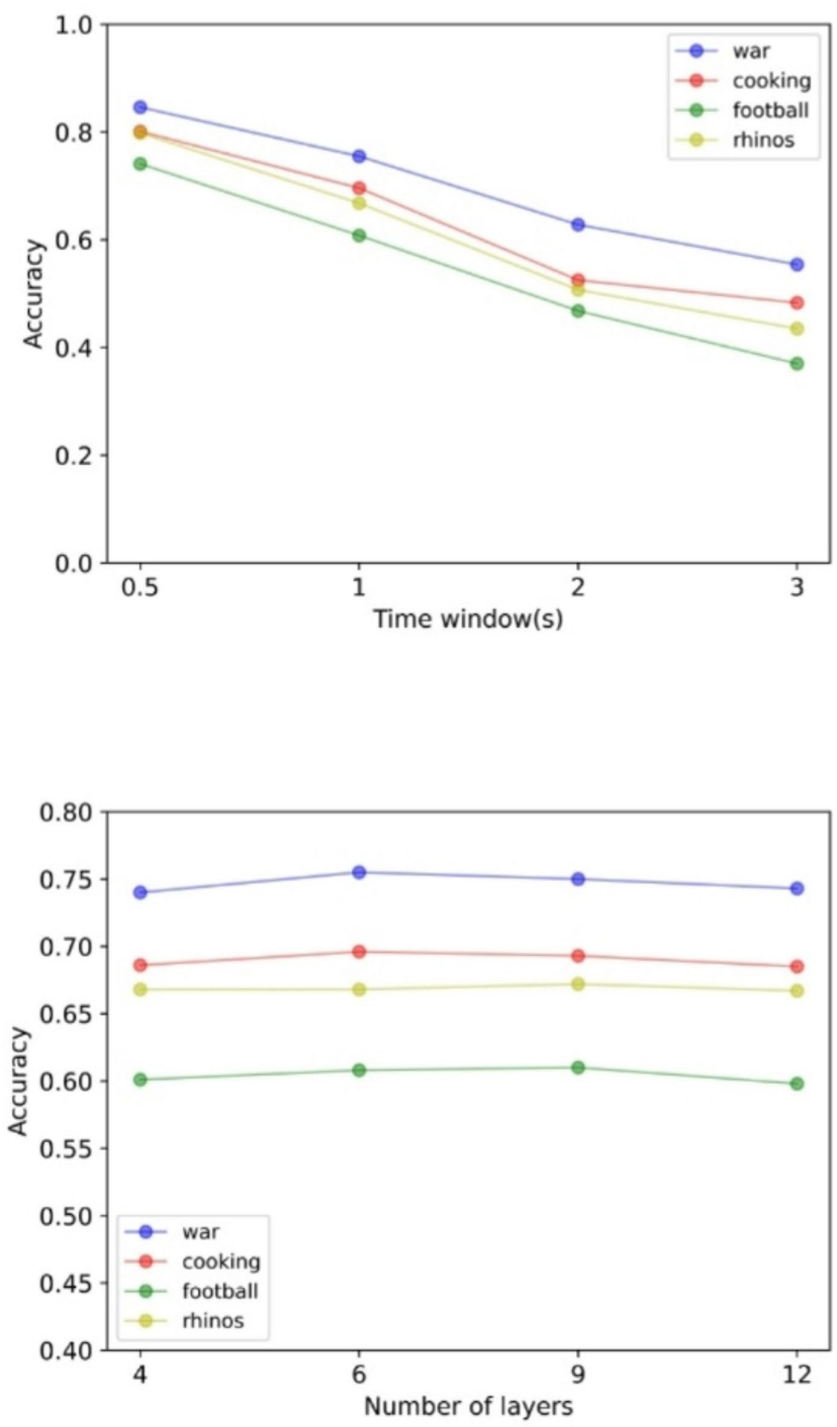
(a) The accuracy of proposed viewport prediction model for different time window predictions. (b) The accuracy of the model for different number of multi-scale Transformer blocks. Testing using Cross Attention module and Fusion module with the same number of layers, i.e. N = K.

To evaluate the model’s robustness over extended prediction horizons, we conducted tests with progressively longer time windows. As evidenced by Fig. 7(a), our model maintains consistently low tile error even as the prediction window increases, demonstrating stable performance across varying temporal scales.

### Impacts of hyperparameter N and K

To evaluate the effects of transformer block number (hyperparameters N and K), we conducted experiments using different layers of blocks, and the results are shown in Fig. 7(b). The prediction accuracy is not proportional to the number of block layers. Accuracy decays after increasing to 9 layers, and the computational cost increases sharply with the number of layers.

### Impacts of new users and video content

**Fig. 8:**
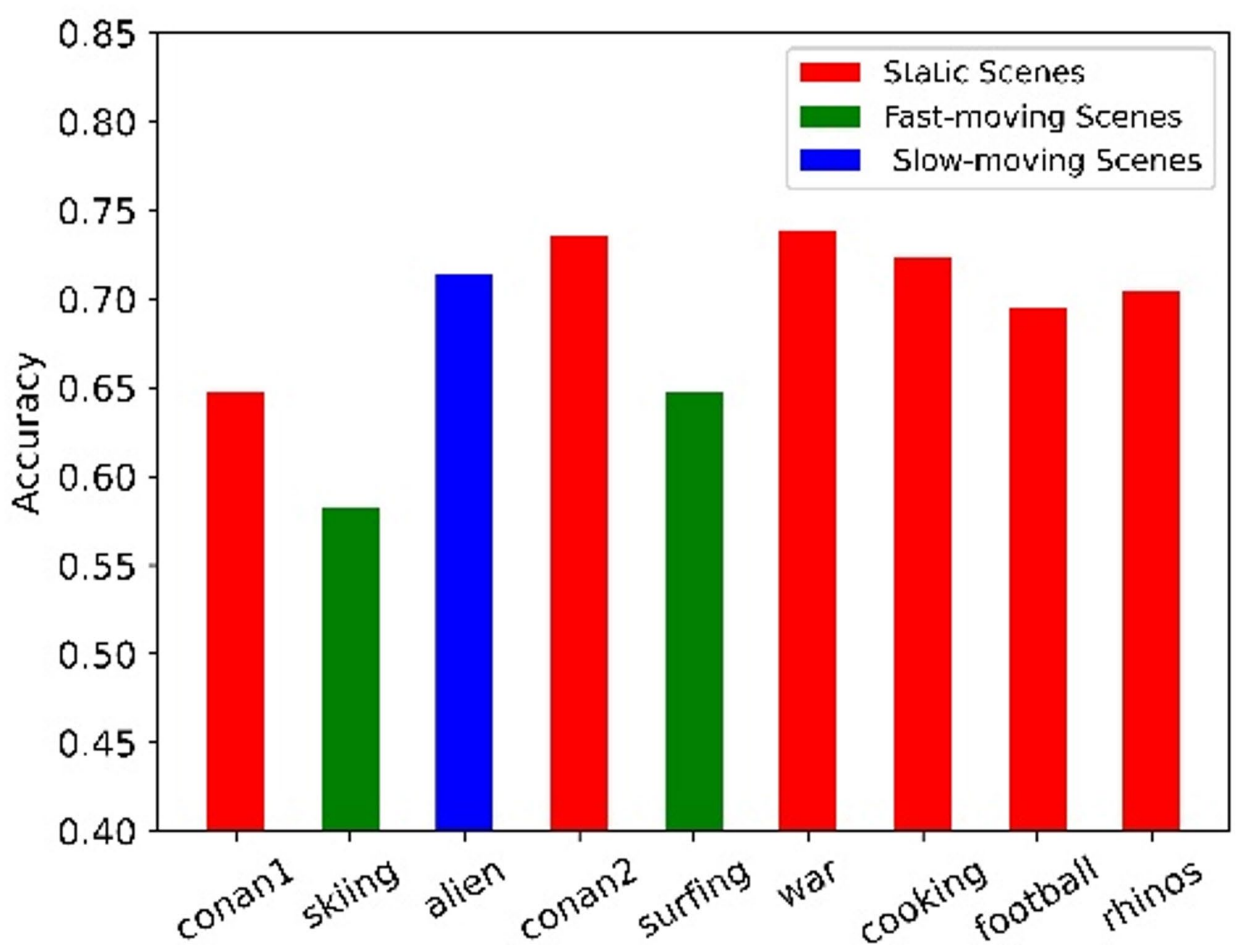
The accuracy of proposed viewport prediction model under different videos.

To understand how the proposed model would adapt to new users for viewport prediction. We retrain the proposed model on 9 videos from^17^, using the data of 40 users and the rest data of 8 users is used as novel data for validation. We show the accuracy of each video in Fig. 8. Despite the fact that the new user’s data has not been trained, our proposed model is still able to achieve a high accuracy rate for the learned videos. The nine videos can be divided into three categories: static scenes with a few scene switches (red), fast-moving scenes (green) and slow-moving scenes (blue)^16^. It can be seen that our proposed model performs best in static scenes. Prediction accuracy decreases in fast-moving scenes. This may be due to the fact that users’ attention shifts more quickly and less user fixation in fast-moving scenes.

## Discussion

This study investigates efficient 360-degree video streaming, addressing the challenges of high bandwidth requirements and dynamic user viewports. While prior research has explored viewport prediction using video content and user trajectories, they have not explicitly addressed the users’ viewing modes and the dependencies between different modalities. As demonstrated in Study^23^, viewport prediction accuracy is substantially influenced by both the stochastic nature of head trajectories and their temporal continuity. Modelling user preferences through visual saliency with user trajectories can indeed improve some viewport prediction accuracy. However, the improvement in accuracy is limited because how to balance the unique user preferences with the visual saliency features is still an issue that requires further research. Future research directions should investigate: (a) more sophisticated preference-saliency integration methods, and (b) the incorporation of multimodal stimuli (e.g., audiovisual cues) to better capture explicit user preferences.

## Conclusion

In this work, we develop a Cross Modal Multiscale Transformer (CMMST) for viewport prediction in 360-degree video streaming. CMMST encodes both user historical trajectories and video content into a unified embedding space, leveraging Transformer architecture to extract Cross Modal spatiotemporal dependencies. The model predicts future viewports by learning user preferences from behavioral patterns and video content characteristics. Experimental results demonstrate the method’s effectiveness, with significant improvements in prediction accuracy over baseline approaches.

## Data Availability

All datasets are publicly released and exempt from human-subjects review. The datasets used during the current study available from the corresponding author on reasonable request.
